# Comparative Quantification of the Phenolic Compounds, Piperine Content, and Total Polyphenols along with the Antioxidant Activities in the *Piper trichostachyon* and *P. nigrum*

**DOI:** 10.3390/molecules27185965

**Published:** 2022-09-13

**Authors:** Jameel Mohammed Al-Khayri, Vinayak Upadhya, Sandeep Ramachandra Pai, Poornananda Madhava Naik, Muneera Qassim Al-Mssallem, Fatima Mohammed Alessa

**Affiliations:** 1 Department of Agricultural Biotechnology, College of Agriculture, Food Sciences, King Faisal University, Hofuf 31982, Al-Ahsa, Saudi Arabia; 2 Department of Forest Products and Utilization, College of Forestry, University of Agricultural Sciences, Dharwad 581401, Karnataka, India; 3 Department of Plant Pathology, and Microbiology, Advanced Teaching and Research Building, 2213 Pammel Drive, Ames, IA 50011, USA; 4 Department of Entomology, Advanced Teaching and Research Building, 2213 Pammel Drive, Ames, IA 50011, USA; 5 Department of Botany, Rayat Shikshan Sanstha’s Dada Patil Mahavidyalaya, Karjat 414402, Maharashtra, India; 6 Department of Botany, Karnatak University, Dharwad 580003, Karnataka, India; 7 Department of Food Sciences and Nutrition, College of Agriculture and Food Sciences, King Faisal University, Hofuf 31982, Al-Ahsa, Saudi Arabia

**Keywords:** *Piper nigrum*, *Piper trichostachyon*, piperine, RP-UFLC, polyphenols, antioxidants

## Abstract

India is the largest producer in the world of black pepper and it is the center of origin for *Piper*. The present study gives a comparative account of the chemical composition of the *Piper nigrum* and its wild putative parent the *P. trichostachyon*. Microextractions were performed and the quantification of six phenolic compounds (namely epicatechin, gallic acid, catechol, chlorogenic acid, caffeic acid, and catechin), piperine from leaves, petioles, and the fruits of both the species, were accomplished using the RP-UFLC system. The polyphenols (phenolic, flavonoid) and their antioxidant activities were also estimated. Among the six phenolic compounds studied, only three were detected and quantified. The polyphenol content correlating to the antioxidant activities was higher in the *P. trichostachyon,* whereas the piperine content was 108 times greater in the *P. nigrum* fruits. The *Piper trichostachyon* comparatively showed a higher content of polyphenols. The microextractions reduced the solvent consumption, the quantity of the plant material, and the amount of time used for the extraction. The first report on the TPC, TF, and the antioxidant activity of the *P. trichostachyon* has been described, and it also forms a scientific basis for its use in traditional medicine. The petioles of both species are good sources of phenolic compounds. A quantitative chemical analysis is a useful index in the identification and comparison of the species.

## 1. Introduction

*Piper* is an economically important genus in the family Piperaceae. The genus *Piper* is represented by approximately 110 species in India, out of which 13 are found in the Western Ghats [1]. The Western Ghats is considered as the center of origin for black pepper [2,3]. The *Piper nigrum* L., is acknowledged as the king of spices. It is an important commodity for agricultural trade [4,5]. Black pepper is well known for its use in food, as a spice, for its use in the cosmetic industry, and for its use as medicine worldwide. Every part of the *P. nigrum* (fruit, seed, leaf, and root) is used in order to treat a number of human and animal disorders including the common cold, cough, various skin disorders, diabetes, hypertension, respiratory disorders, etc., in non-codified and codified systems of traditional medicine. [6,7,8].

Traditionally an ancestral species, the *Piper trichostachyon* (pouched pepper) was reportedly used to treat similar conditions as the *P. nigrum*, by folk healers in India [3,4,9,10,11]. According to folklore, the fruits of the *P. trichostachyon* were collected in the wild and then used in folk medicine to treat coughs, colds, fevers, migraines, toothaches, as well as to increase sperm count [7]. Folk healers in the Belagavi region used the entire leaf for a faster recovery, following child birth and also to treat respiratory tract infections, (field observations). They claim that the *P. trichostachyon* was better and faster than the *P. nigrum* plant for the treatment of various ailments.

The *Piper nigrum* reportedly consists of diverse bioactive alkaloids, sterols, fatty acids, terpenes, amides, and other phytoconstituents that are responsible for various biological activities [8]. However, there are no scientific studies available on the biological activities of the *P. trichostachyon*, although it was traditionally used.

Reports indicate that piperine is the major marker alkaloid component present in the *P. trichostachyon* and it is reported to be responsible for the biological activities shown by black pepper. Piperine possesses a range of pharmacological properties and reports also indicate its in vivo and in vitro antioxidant capabilities [5,6,12,13,14,15,16,17,18]. The presence of piperine and a few other phytoconstituents are found in the *P. trichostachyon;* but fewer studies have been carried out concerning its phenolic profiles, piperine content, and polyphenols in correlation to the antioxidants in the leaf, petiole, and the fruits [11,19]. Although the chemical profiling and biological activity of the *P. nigrum* was previously studied, an attempt was made to make comparisons between the two species which are used in folk medicine. The plant polyphenols are a widely distributed class of phytochemicals in the floral kingdom and presently they are widely studied in order to find out more about their antioxidant capabilities [20]. Hence, the present study has analyzed a few common plant polyphenols in order to understand the antioxidant capabilities of both *Piper* species.

## 2. Results

The results related to the TPC and TF are represented in the Table 1. The contents were determined using a regression equation for the respective calibration curves (TPC: y = 0.0017x − 0.0669; *R*^2^ = 0.9426 and TF: y = 0.0049x − 0.0028; *R*^2^ = 0.9892). All of the results are on a fresh weight basis.

### 2.1. Estimation of the TPC

The total phenolic content of the *P. trichostachyon* was greater than that of the *P. nigrum* (Table 1). The highest TPC was observed in the petioles (*P. trichostachyon* 13.01 ± 0.65 mg TAE/g FW and *P. nigrum* 7.50 ± 0.38 mg TAE/g FW) of the respective species followed by the fruits and leaves, respectively. The *Piper trichostachyon* fruit extract showed a content of 11.13 ± 0.56 mg TAE/g FW, whereas the *P. nigrum* fruit extracts showed a lower amount (6.71 ± 0.34 mg TAE/g FW) of phenolic content.

### 2.2. Estimation of the TF

The leaves showed equal quantities of flavonoids (Table 1) in the *P. trichostachyon* (5.14 ± 0.26 mg QE/g FW) and the *P. nigrum* (5.61 ± 0.28 mg QE/g FW) than in the other parts tested. It was interesting to observe that the fruits of the *P. nigrum* (63.11 ± 3.16 mg QE/g FW) had a ~30-fold higher quantity of flavonoids than the fruits of the *P. trichostachyon* (2.19 ± 0.11 mg QE/g).

### 2.3. Antioxidant Activities

The petiole extracts of the *P. trichostachyon* and the leaf extracts of the *P. nigrum* exhibited a greater DPPH radical scavenging activity than other parts (Table 1). The concentration of 10 mg/mL petiole extracts of the *P. trichostachyon* reached a scavenging activity of ~641.00 µM AEAC and a similar concentration. The leaf extract of the *P. nigrum* had a 496.50 µM AEAC scavenging activity. The DPPH radical scavenging activities of all of the extracts was found to be comparable with the ascorbic acid equivalent capacity.

The ferric reducing ability is a widely used assay in the evaluation of the antioxidant potential in dietary polyphenols [16]. The reducing ability of the different parts of the *Piper* species ranged from 116.50 to 474.50 µM AEAC. The greater ferric reducing activity was demonstrated by the petiole extracts in both species, followed by the leaves and fruits, respectively (Table 1).

### 2.4. Quantification of the Phenolic Compounds and Piperine Using the RP-UFLC Method

**Figure 1:**
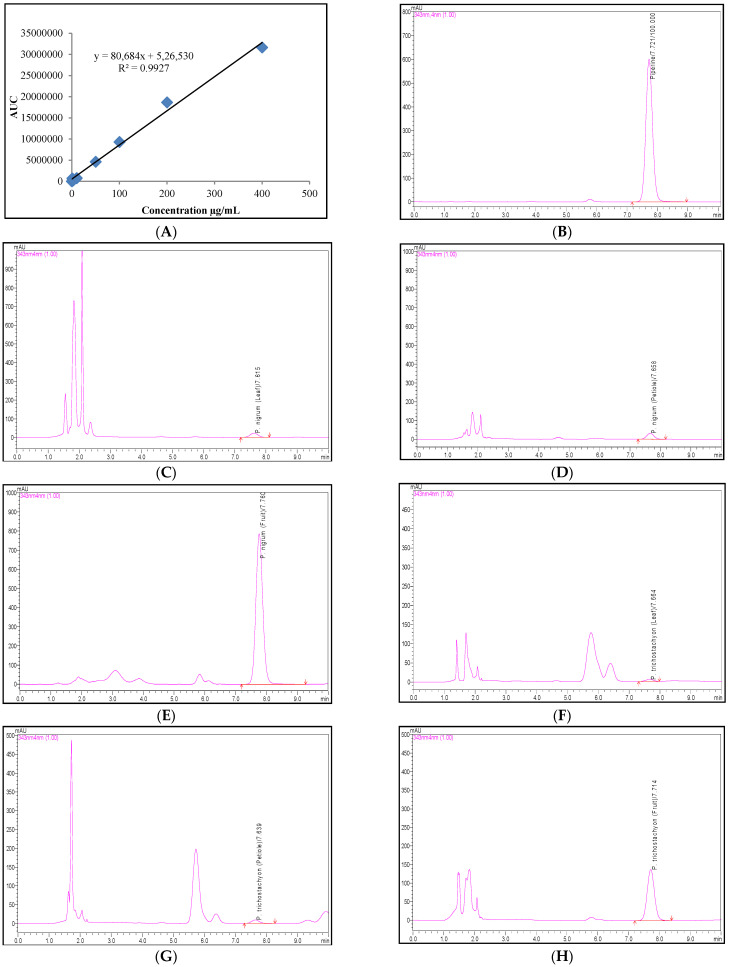
(**A**) Calibration curve of standard piperine; UFLC profiles of (**B**) 100 µg/mL of standard piperine; (**C**–**E**) *P. nigrum*; (**F**–**H**) *P. trichostachyon* (**C**,**F**) leaf extracts; (**D**,**G**) petiole extracts; (**E**,**H**) fruit extracts. **Size:** Column width.

**Figure 2:**
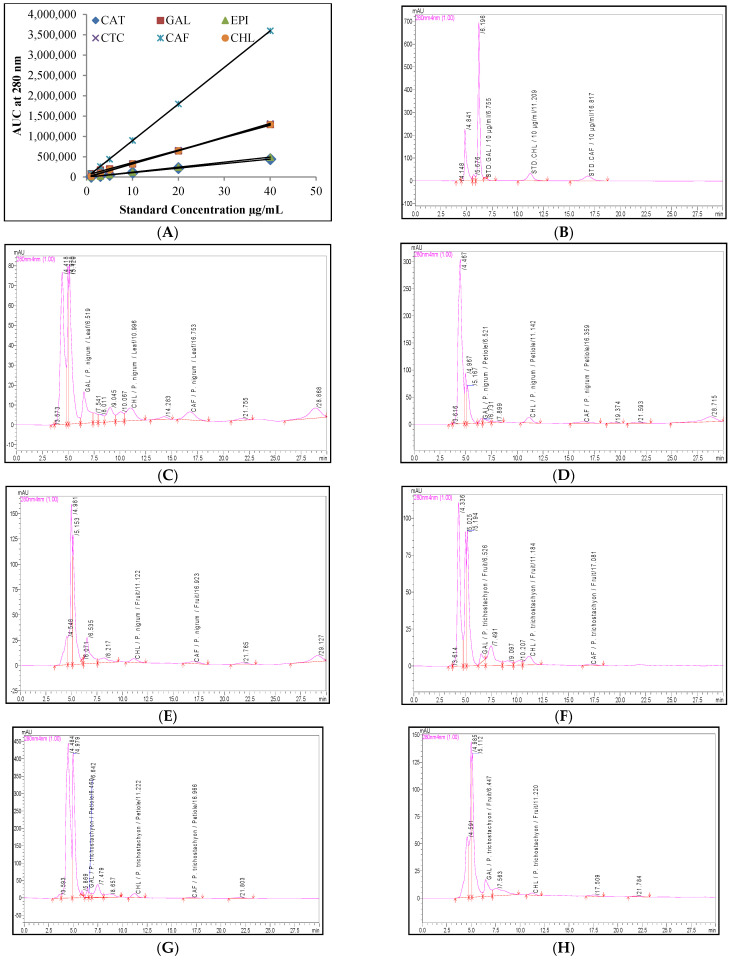
(**A**) Four point calibration curves for all six standards (CAT, GAL, EPI, CTC, CAF, and CHL); (**B**) UFLC profile of the standards (10 µg/mL): gallic acid (GAL), chlorogenic acid (CHL), and caffeic acid (CAF) detected in different parts of the two *Piper* species; UFLC profiles of (**C**–**E**) *P. nigrum*; (**F**–**H**) *P. trichostachyon*; (**C**,**F**) leaf extracts; (**D**,**G**) petiole extracts; (**E**,**H**) fruit extracts. **Size:** Column width.

The quantitative determination of the six phenolic compounds and piperine was accomplished using the RP-UFLC technique. The study results were represented as mg/100 g on a fresh weight basis (Table 2). The calibration curves were constructed from six concentrations of six standard phenolic compounds and eight concentrations of standard piperine against their respective area under the curve (AUC) with the coefficient of determination (*R*^2^) above 0.975 (Table 2, Figure 1A and Figure 2A). The regression equation showed a significant relationship between the peak areas and the concentrations (Figure 1A and Figure 2A). The regression equation was used in order to estimate the respective chemical content from the various extracts obtained from the different parts of both *Piper* species. The limit of detection (LOD) and the LOQ for the phenolic compounds and piperine are presented in Table 2. The lowest calibrators used during the study were1 µg/mL for the phenolic compounds, and 0.01 µg/mL for piperine. Less than 2% of the RSD values indicate this method to be precise and reproducible (Table 2). The method validation was carried out by spiking 50 µL (100 µg) of standard piperine to an equal volume of the *P. trichostachyon* petiole extracts in order to acquire a recovery within the range of 95–100%.

The retention times and other attributes, including the LOD, LOQ, and tailing factor, related to the RP-UFLC run for the selected phenolic compounds are presented in Table 2. All of the standard runs produced clear and sharp peaks without any ambiguity in the identification. Of the six phenolic compounds tested using the RP-UFLC analysis for the two species of *Piper*, only three were detected and quantified. The chlorogenic acid, gallic acid, and caffeic acid were detected; catechin, epicatechin, and catechol were not detected.

The content of both the gallic acid (6.53 ± 0.33 mg/100 g) and the caffeic acid (0.96 ± 0.05 mg/100 g) were high in the leaf extract of the *P. nigrum,* whereas chlorogenic acid (3.64 ± 0.18 mg/100 g) was high in the petiole extracts of the *P. nigrum*. The caffeic acid remained undetected in the *P. trichostachyon* fruit extracts and the same was observed regarding the gallic acid in the *P. nigrum*. 

The UFLC profiles with a retention time of 7.730 ± 0.09 min for the detection of piperine in the samples were obtained. Sharp and distinct standard peaks confirmed the purity (98%) and reduced the compatibility questions between the extraction solvent and the mobile phase in the investigation. The auto-scaled chromatograms were created for standard piperine, fruit, petiole and leaf extracts of the *P. nigrum,* and the *P. trichostachyon* (Figure 1B–H). At a 20% concentration, the *P. nigrum* fruit extract produced broad and flat-topped piperine peaks. Further dilution of the extract to 1.0% (Figure 1E) resolved this issue. When compared to other parts of the plant, both fruit species contained the greatest amount of piperine. The piperine content in the *P. nigrum* fruits (1555.50 ± 77.80 mg/100 g) was higher (108 times) than in the *P. trichostachyon* fruits (14.40 ± 0.80 mg/100 g). A lower piperine content was found in the petioles, followed by the leaves in both species, respectively (Table 3).

The data represented In Table 1 and Table 3 were subjected to statistical analyses using the two factor ANOVA. The results are presented in Table 4. It can be inferred from the results that there exists a significance within the columns i.e., various phytochemical tests and antioxidants with a *p* value < 0.05.

## 3. Discussion

The main purpose of this study was to assess and compare the phenolic compounds, piperine, and antioxidant activities in the leaves, petioles, and fruits of the *P. nigrum* and *P. trichostachyon*. In this study, the TPC in the leaves, petioles, and in the fruits of both species ranged from 6.26 mg TAE/g to 13.01 mg TAE/g. Though there was some variation in the phenolic content in both species, the pattern of content in the different parts remained the same. The *P. trichostachyon* fruit showed a greater amount of phenolic content than the leaf, whereas the *P. nigrum* fruit showed the lowest amount of the TPC. However, Ashadevi et al. found that the TPC was 5.04 mg GAE/g, using the methanolic extracts of the pepper [21]. Nahak and Sahu reported 62.3 ± 0.08 µg/g of the TPC in the ethanol fruit extract of the *P. nigrum* [22]. The TPC in black pepper (3.0 mg/g GAE) discussed in the earlier study by Shan et al. [23] was less than that observed during the present investigation. 

The higher levels of flavonoids compared with the phenolic content were determined in the *P. nigrum* fruits. These results coincide with those of Ashadevi et al. [21]. It is also reported that the polyphenols in green pepper vary from that of black pepper [24].

In general, the genus *Piper* is valued for its fruits for various uses, including in medicine [8]. However, Salehi et al. reported that the traditional use of 106 different *Piper* species that are found all over the globe, and in particular 90% of these species, the different plant parts including the roots, leaves, stems, bark, and branches are used as well as the fruits and seeds [12]. The fruits and seeds of the *P. nigrum* are the most used parts of the plant, hence they are considered for maximum use in biological activities [8]. It has been reported that until 2018, a total of 17 antioxidant assays, among which 13 are in vitro and four are in vivo studies [8]. Several in vitro assays were carried out, based on different radical scavenging activities (namely, the DPPH, ABTS, nitric oxide, superoxide anion, hydroxy, hydrogen superoxide anion, and hydrogen peroxide radical scavenging). Other assays including the FRAP, ORAC, linoleic acid and lipid peroxidation, phosphomolybdenum assay, as well as the total antioxidant activities, were performed in order to measure the antioxidant capacity in *P. nigrum* fruits [8]. In this study, the antioxidant activities were assessed using the DPPH and FRAP methods and the findings were in line with studies carried out by Nahak and Sahu, as well as those by Prasad et al. [22,25]. It has been reported that 3.83 mg/g of the total phenolic content in black pepper fruits, which contains 4.7 mmol/100 g of the FRAP antioxidant contents and is responsible for 19.5% of the DPPH inhibitory antioxidant activity [26]. A review by Yashin et al. summarizes the antioxidant activities, the total polyphenols, and the flavonoids of the various spices and culinary herbs. They pointed out the positive correlation between the antioxidant capacities and the corresponding total polyphenols in some spices, including black pepper [27]. The oil extracted from black pepper was also evaluated for the TPC, TF, and antioxidant activity [28]. Shanmugapriya et al. estimated the antioxidant ability of the *P. nigrum* leaves using acetone, ethyl acetate, and aqueous extracts. The ethyl acetate extract exhibited the highest DPPH scavenging properties, whereas the water extract was an able ferric reducer [29].

The greater antioxidant activity observed in the petioles can be attributed to a higher phenolic content. All of the in vitro methods studied earlier showed the effective antioxidant ability of the *P. nigrum* plant. The present comparative study shows a higher amount of antioxidant activity on the part of the *P. trichostrachyon* plant compared with the respective parts of the *P. nigrum*. Nearly 2.5 times greater antioxidant activity was seen in the fruits of the *P. trichostachyon* over the *P. nigrum*. Antioxidants are generally correlated for their active involvement in reducing cough, cold, and other respiratory problems. The use of the *P. trichostachyon* fruits in the treatment of colds, coughs, and fevers in traditional medicine is corroborated by the presence of antioxidants.

A higher amount of the TPC corresponded with a higher amount of chlorogenic acid detected in the petioles of both species. According to Gulçin, the antioxidant activity of black pepper is owed to the presence of phenolic compounds [30], wherein Zarai et al. correlated the antioxidant activities to piperine and piperic acid [31]. Nakatani et al. used the ferric thiocyanate and thiobarbituric acid methods, in order to study the antioxidative capacity of piperine and five amides from the *P. nigrum*. They reported that piperine had shown no activity while the phenolic amides showed significant activity and therefore they accounted the phenolic complexes for the antioxidant activity in black pepper [32]. However, it is difficult to make this conclusion, pertaining to the specific phenolic compound as well as to correlate it with the total phenolic content and antioxidant activities presented herein. The two factor ANOVA also indicated the significance of variation between the test results for total phenolic content and antioxidant activity.

Piperine (1-peperoyl piperidine) is an alkaloid responsible for the spicy nature of black pepper. Piperine has been found in many domesticated cultivars and wild species of the *P. nigrum* [12,14,16,33] and range from 2% to 7.4% [15]. Smilkov et al. discussed the role of piperine as a nutraceutical over aromatic spice [14]. This new role of piperine was mainly substantiated by its diverse biological activities but not limited to antioxidant [13,34], anticancer [35], anti-inflammatory [36], antimicrobial, immunomodulatory, antihypertensive and antiplatelet [3], antiasthmatics [5], analgesic, antispasmodic, antipyretic, anti-diarrheal, antidepressants, anxiolytic, and hepatoprotective activities [6,18]. Piperine is also reported to improve fertility and cognitive activities [37]. This marker compound in black pepper acts as an oral bioavailability enhancer agent. It increases the therapeutic efficacy of several drugs, nutrients, and vaccines by inhibiting various metabolizing enzymes [38]. A greater use and popularity of the *P. nigrum* over the *P. trichostachyon* in folk medicine can be related to the presence of a higher the amount of piperine. The presence of piperine is reported by Upadhya et al. in the *P. trichostachyon*. The comparative study results between the same two species are similar [11]. The phytochemical assessments reported the presence of trichostachin, cyclo stachin, and lignin amides in the stem and leaf parts of the *P. trichostachyon*. Kaul et al. testified to the absence of such chemical compounds in the fruits of the *P. trichostachyon* [39]. Pouched pepper showed good antioxidant capabilities over black pepper, thereby indicating that the presence of a higher amount of piperine is not the only phytochemical component responsible for all biological activities reported.

The phytochemical evidence is also used in the understanding of phylogenetic relationships in taxonomy and also as a tool for quality standards in food and pharma industries [40]. The results of the present work indicate a comprehensive understanding of the marker of piperine distribution in leaves, petioles, and fruits of the *P. nigrum* and the *P. trichostachyon,* which will help in its identification.

Piperine is the principal amide extracted from pepper using various extraction methods such as the super fluid critical, Soxhlet, and microwave-assisted extractions, refluxing, sonication, continuous shaking methods, accelerated solvent, and the Naviglio Extractor [15,31,41]. Unlike the above techniques, the microextraction method requires a small amount of plant material with a lower solvent intake, thus making it suitable for the analysis of larger sample sizes and speedy analyses. The studies on the wild relatives of a crop, such as the *Piper*, are also of great importance in order to find the source of useful genes and the present comparative studies on the two species of *Piper* may add to the understanding of their evolutionary relationships [3,42]. It is evident that the piperine content in pouched pepper has no comparison with that of black pepper. The higher content of piperine in the *P. nigrum* has likely been achieved over the course of domestication, whereas the *P. trichostachyon* responded to natural selection and adaptations. Hence, it is observed that the phenolic and flavonoid contents were higher in the *P. trichostachyon* than in the *P. nigrum*.

Further chemical and pharmacological investigations are recommended in order to evaluate the potential of the *P. trichostachyon* species and to validate its medicinal uses. 

## 4. Materials and Methods

### 4.1. Standards and Chemical Reagents

All of the standards (HPLC grade), quercetin, and tannic acid were procured from Sigma–Aldrich (India). The solvents used (Water and methanol) were of HPLC grade. The DPPH, 2,4,6-Tris(2-pyridyl)-s-triazine, Folin–Ciocalteu’s phenol reagent, aluminum chloride, glacial acetic acid, hydrochloric acid, and other chemicals were of analytical grade.

### 4.2. Plant Material

Leaf, petiole, and fruit material of the *P. nigrum* and the *P. trichostachyon* mature plants were collected from the Belagavi (15.63834° N, 074.27841° E) and Uttara Kannada (14.7853° N, 074.7757° E) regions of the Western Ghats in Karnataka state, India, respectively. The collected specimens were botanically identified and authenticated by Dr. Harsha Hedge, by comparing the submitted herbarium sheets from the herbaria of the ICMR-National Institute of Traditional Medicine, Belagavi, Karnataka, India (Voucher Numbers—*P. nigrum*: RMRC 1213 and *P. trichostachyon*: RMRC 1214).

### 4.3. Sample Extraction

The previously described microextraction method by Ankad et al. was deployed [43]. Three individual fresh samples of both species were collected and pooled for extraction. The leaf, petiole, and fruit material (0.3 g each) were separately and thoroughly mixed in microcentrifuge tubes using a micro-pestle in 1.5 mL methanol. This mixture was extracted on a hot water bath at 60 ± 2 °C for 5 min, cooled to room temperature, and centrifuged at 5000 rpm for 15 min in order to obtain a supernatant (20%). These extracts were directly used in order to analyze the phytoconstituents using the RP-UFLC method, where they were diluted to 1% for the quantitative determination of the polyphenols, and then assessed for their antioxidant activities.

### 4.4. Estimation of the Total Phenolic Content (TPC)

The total phenolic content was determined using Folin–Ciocalteu’s phenol reagent mentioned by Pawar et al. [44]. The λmax was set to 760 nm and the absorbance of blue color was read, using distilled water instead of standard tannic acid in the reaction mixture (250 µL), as blank on a Thermo Fischer’s, Multiskan GO UV/Vis microplate spectrophotometer. Similarly, 1.0% extracts were evaluated in order to express the mg tannic acid equivalent per gram of fresh weight.

### 4.5. Estimation of the Total Flavonoid (TF)

The method described by Luximon-Ramma et al. [45] for the total flavonoid content was used in order to quantify the TF [45]. The λmax was set to 368 nm and the optical density was measured and assessed with 2% aluminum chloride as blank for the samples. The results obtained for the different concentrations of the standard quercetin were expressed as the mg quercetin equivalent per gram of fresh weight.

### 4.6. Antioxidant Activities

#### 4.6.1. 1,1-Diphenyl-2-picrylhydrazyl (DPPH) Radical Scavenging Assay

The method explained by Brand–Williams et al. was deployed in order to measure the DPPH radical scavenging antioxidant activity [46]. The method suggests setting the λmax at 515 nm in order to measure the methanol as blank and the decline in pink color produced in the standard concentrations and samples. The concentrations of ascorbic acid were used as a reference standard during the experiment. The observations were expressed as µM AEAC (ascorbic acid equivalent capacity).

#### 4.6.2. Ferric Reducing Antioxidant Power (FRAP) Assay

The FRAP method given by Benzine and Strain was used in order to determine the total antioxidant power of the extracts [47]. The resultant reactants were read at 593 nm, and the obtained readings were conveyed as µM AEAC.

### 4.7. Quantification of the Phenolic Compounds and Piperine Using the RP-UFLC Method

#### 4.7.1. Instrumentation and Conditions

A Shimadzu chromatographic system (Model no. LC-20AD) with a dual λ UV absorbance diode array detector (SPD-M20A) was used for the RP-UFLC analysis. The data processing was performed using a built-in LC-Solution software system. The separation of the compounds was achieved on a Qualisil BDS 250 × 4.6 mm (5 µm) C18 column for phenolic compounds and a Hibar 250 × 4.6 Lichrospher 60 RP-select B (5 µm) column for the piperine. Acetonitrile, water, and glacial acetic acid (with a ratio of: 12:85:3) was used as a solvent system for the separation of the phenolics, whereas piperine was separated using water and methanol (30:70) in an isocratic mode with a common injection volume (20 µL). The pressures were maintained by adjusting the flow rates (0.7 and 1.4 mL/min) at a λmax of 280 nm and 343 nm in order to detect the phenolic compounds and piperine, respectively.

The calibration, linearity, LOD, LOQ, retention time, and the tailing factor parameters according to the ICH guidelines for the phenolic compounds and piperine are provided in Table 2. The standard signal/noise methods and parameters were used in order to perform the detection and quantification limits (LOD and LOQ) [11,41].

#### 4.7.2. System Suitability

Three replicate injections of the standards at a specific concentration were injected in order to determine the suitability of the system (phenolic compounds: 3 µg/mL; piperine: 50 µg/mL). The resolution and tailing factors were repeatedly determined by the peak shape and area for the standards tested.

### 4.8. Statistical Analysis

The figures obtained as data for the experiments were expressed as the mean of three separate readings alongside its RSD. The tables were created using Microsoft Excel 2019 and the statistical analysis was performed using the Graph Pad Instat, statistical software (Ver. 3.06). The two-factor analysis of variance (ANOVA) without replication between the contents of Table 1 and Table 3 was established using Microsoft Excel.


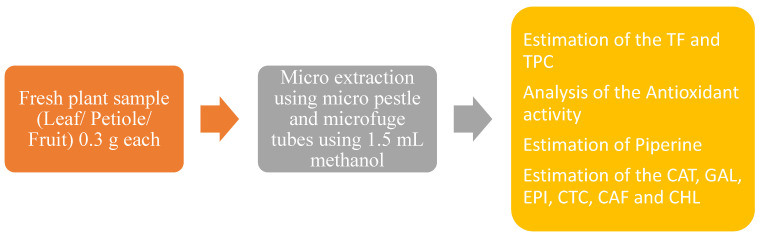


**Graphical representation Methodology**.

## 5. Conclusions

The *Piper trichostachyon* has higher phenolic content corresponding to higher antioxidant activity than the *P. nigrum,* which supports its use in folk medicines. The petioles of both species were a good source of phenolic compounds. Piperine was quantified by the RP-UFLC analysis in the leaf, petiole, and fruit of both species. The content of piperine was higher in the *P. nigrum* compared with the *P. trichostachyon* fruits. The quantitative analysis of piperine is a useful index in quality assurance and pharmacognosy research, even at a lower extraction scale. However, detailed phytochemical, safety, and efficacy studies in the *P. trichostachyon* are needed in order to prove its traditional medicinal use over the *P. nigrum*.

## Figures and Tables

**Table 1:** Total phenolic content, flavonoid content, and antioxidant activities of the extracts obtained from various parts of the *P. nigrum and P. trichostachyon*.

Species	Part	TPC mg/g	TF mg/g	DPPH µM	FRAP µM
***P. nigrum***	**Leaf**	6.26 ± 0.31	5.61 ± 0.28	496.50 ± 24.82	151.00 ± 07.55
	**Petiole**	7.50 ± 0.38	1.89 ± 0.09	366.00 ± 18.33	300.50 ± 15.03
	**Fruit**	6.71 ± 0.34	63.11 ± 3.16	85.50 ± 4.28	116.50 ± 05.83
***P. trichostachyon***	**Leaf**	7.28 ± 0.36	5.14 ± 0.26	117.00 ± 5.85	181.00 ± 09.05
	**Petiole**	13.01 ± 0.65	3.42 ± 0.17	641.00 ± 32.05	474.50 ± 23.73
	**Fruit**	11.13 ± 0.56	2.19 ± 0.11	176.50 ± 8.83	181.00 ± 09.05

Values in the table represent the fresh weight of the respective samples; results of the DPPH and FRAP are represented as µM AEAC (ascorbic acid equivalent antioxidant capacity).

**Table 2:** Standard parameters and result attributes of RP-UFLC analysis.

Parameters	Units/Abb	Alkaloid	Phenolics					
		PIP	CAT	GAL	EPI	CTC	CAF	CHL
**Dissolved in**	**mg/mL**	MeOH	MeOH	MeOH	MeOH	MeOH	MeOH	MeOH
**Concentration**	**µg/mL**	0.01–400	1–40	1–40	1–40	1–40	1–40	1–40
**Total levels**	**--**	8	6	6	6	6	6	6
**Linearity equation**	**--**	y = 8068x + 5265	y = 11,117x − 2913	y = 30,739x + 47,889	y = 12,349x − 4444	y = 33,185x − 8782	y = 90,138x − 7086	y = 32,301x − 498.7
***R^2^***	**--**	0.992	0.999	0.997	0.999	0.999	0.999	0.999
**LOD**	**ng/mL**	120.00 ± 60.00	17.80 ± 0.00	82.40 ± 0.02	40.92 ± 0.02	21.31 ± 0.00	5.00 ± 0.02	36.90 ± 0.03
**LOQ**	**ng/mL**	350.00 ± 20.00	54.00 ± 0.01	249.70 ± 0.09	124.01 ± 0.05	64.60 ± 0.02	15.16 ± 0.01	111.97 ± 0.04
**Retention time**	**min**	7.769 ± 0.090	11.919 ± 0.146	6.208 ± 0.040	16.056 ± 0.158	12.455 ± 0.037	15.918 ± 0.056	11.670 ± 0.245
**RT RSD**	**%**	1.161	1.224	0.643	0.982	0.297	0.351	2.100
**Theoretical Plates**	**N**	5320.8 ± 376.8	5112.8 ± 398.9	6684.3 ± 241.1	4796.7 ± 299.1	5467.4 ± 331.5	4610.1 ± 254.6	2305.1 ± 397.9
**Tailing factor**	**Tf**	1.103 ± 0.010	0.897 ± 0.106	1.049 ± 0.024	0.803 ± 0.029	1.107 ± 0.044	0.950 ± 0.040	1.205 ± 0.071

**Table 3:** Content of piperine (mg/100 g) and the six phenolic compounds from various parts of the *P. nigrum* and the *P. trichostachyon*, as determined using the RP-UFLC analysis.

Plant	Parts	Piperine (mg/100 g)	Phenolic Compounds Contents (mg/100 g)					
			CAF	GAL	CAT	CTC	CHL	EPI
***P. nigrum***	**Leaf**	2.58 ± 0.13	1.10 ± 0.06	5.64 ± 0.28	ND	ND	4.08 ± 0.20	ND
	**Petiole**	3.32 ± 0.17	0.75 ± 0.04	1.05 ± 0.05	ND	ND	4.39 ± 0.22	ND
	**Fruit**	1555.49 ± 77.77	0.56 ± 0.03	ND	ND	ND	1.78 ± 0.09	ND
***P. trichostachyon***	**Leaf**	0.60 ± 0.03	0.21 ± 0.01	0.74 ± 0.04	ND	ND	2.97 ± 0.15	ND
	**Petiole**	0.97 ± 0.05	0.39 ± 0.02	3.11 ± 0.16	ND	ND	4.24 ± 0.21	ND
	**Fruit**	14.35 ± 0.72	ND	5.91 ± 0.30	0.26 ± 0.01	ND	0.84 ± 0.04	ND

ND: Not Detected; CAF: Caffeic acid; GAL: Gallic acid; CAT: Catechin; CTC: Catechol; CHL: Chlorogenic acid; EPI: Epicatachin.

**Table 4:** Two factor ANOVA without replication between the contents of Table 1 and Table 3.

Source of Variation	SS	df	MS	F	*p*-Value	F Crit
**Rows**	184,073.8	5	36,814.75	0.866	0.511	2.422
**Columns**	572,749.9	9	63,638.87	1.497	0.178	2.095
**Error**	1912,891	45	42,508.68			

## Data Availability

Data sharing is not applicable to this article.

## Abbreviations

AEAC: Ascorbic acid Equivalent Antioxidant Capacity; AUC: Area Under Curve; CAF: caffeic acid; CAT: catechin; CHL: chlorogenic acid; CTC: catechol; DPPH: 1, 1 diphenyl-2-picrylhydrazyl; EPI: epicatechin; FRAP: Ferric Reducing Antioxidant Power; GAL: gallic acid; LOD: Limit of Detection; LOQ: Limit of Quantification; ORAC: Oxygen Radical Absorbance Capacity; QE: Quercetin Equivalent; RP-UFLC: Reversed Phase—Ultra Flow Liquid Chromatography; RSD: Relative Standard Deviation; TAE: Tannic Acid Equivalent; TF: Total Flavonoids; TPC: Total Phenolic Content.
